# The Organisation of Panel Practice

**Published:** 1923-02

**Authors:** R. W. Harris

**Affiliations:** (Formerly Assistant Secretary, responsible for the administration of National Health Insurance Medical Benefit, Ministry of Health)


### THE ORGANISATION OF PANEL PRACTICE.

**By R. W. HARRIS (Formerly Assistant Secretary, responsible for the administration of National Health Insurance Medical Benefit, Ministry of Health).**

COMPLAINT has been made by Insurance Committees that they are so largely engaged in carrying out arrangements which have been imposed upon them by the Minister that they are unable to develop the Insurance Medical Service on their own initiative. This is, to a large extent, unavoidable. In a national system the nature of the service required and the machinery for securing it must inevitably be determined centrally and prescribed in some considerable detail in regulations issued by the central department. But the medical service for the insured would be a dismal failure if it were to depend solely upon the written word emanating from Whitehall, or even upon Whitehall control carried out by Government inspectors. This medical service is, and must always remain, a very human service, and the measure of its success will depend upon the extent to which the patient has confidence in his doctor and the extent to which the doctor in turn feels assured that he may look to those engaged in the local administration not only for justice and fair play, but for a sympathetic, human interest in his work.

### Extravagant Demands.

Not only is it true that the success of the service within the limits prescribed by statute or regulation depends upon the spirit in which it is administered locally, but there is also much scope for the improvement of the service on the initiative of the Insurance Committee. We are too apt to think that no development or improvement is possible until a movement is made nationally and more money is forthcoming from somewhere or other. What is still more unfortunate is that when central conferences are held, whether of Approved Societies, Trade Union organisations, Insurance Committees or other bodies interested in the welfare of the insured, the demand which goes forth is for something which is not practical politics. I make an honourable exception for the comparatively modest resolution passed by the National Association of Insurance Committees at their last autumn conference to the effect that there should be a complete laboratory service for the insured, the cost of which would, I believe, prove to be relatively very small. The best is ever the enemy of the good. Improvements in local administration of the medical service might well be arranged by an individual Insurance Committee within the scope of the existing contract without waiting for a national scheme. The particular improvement to which I address myself in this article is that of the organisation of panel practice.

### Improvement by Organisation.

It should not be impossible for an Insurance Committee in a moderately large borough, with the co-operation of the Panel Committee, to bring about a great improvement in the service merely by means of organisation. We all know what happens at present in any average collection of medical practices. This doctor's surgery accommodation leaves much to be desired, though it cannot be said to be either improper or insufficient judged by a not very exacting standard. Another doctor's waiting room accommodation is barely adequate, and is a depressing affair at best. Another doctor lacks proper equipment. Too many, especially in times of pressure, would have to admit a certain amount of muddle in regard to the writing of certificates and prescriptions and the handling of records. And with it all the average doctor is more or less at the beck and call of patients at any hour of the day and night, including Sunday, which for others is a day of rest. We have only to consider this picture soberly to appreciate the need for organisation in the interests of the doctors themselves no less than in that of the service.

### Central Surgeries.

The initiative for bringing about this organisation must clearly come from outside the doctors' own ranks, and a good deal of driving force would be required to get a scheme going. But it would be of the essence of any such scheme that it should be undertaken with the goodwill of the doctors and in the main financed by them. It requires little imagination to see what a tremendous improvement could be made both in the service as a whole and in the conditions of life of the doctors themselves if they were to agree to establish central surgeries and waiting rooms well lighted, well equipped and comfortable, where the assistance of clerks and dispensers would be available in the handling of certificates, prescriptions and records, and where surgeries could be taken in rotation. The doctors would stand to gain by the sharing of the cost of the more expensive equipment, the opportunities of consultation with one another on difficult cases and on medical matters generally, the regular facilities for deputising arrangements, the golden hours free from demands by patients, and the relief from much of the clerical and preliminary work which would follow from such effective organisation, with a consequent concentration of the doctors' time and energies on their real job.

### The Gain to the Patients.

The insured persons, also, would gain all along the line. With such an organisation in existence, the provision of nursing and dentists, of specialist services and the sortation of cases for hospital treatment would be greatly facilitated when the financial problem was solved. I commend the first step, however, the organisation of panel practice, to the notice of some Clerk to an Insurance Committee who has won the confidence of the doctors of his area and has vision combined with common sense and organising capacity. It is not going to be an easy task to surmount the prejudice and suspicion of some, at any rate, of the doctors. But I believe it can be done. Nor is the question of the Insurance Committee giving assistance from their Administration or General Purposes Funds by any means to be ruled out.

